# Intra- and interspecific variation of *Amblyomma* ticks from southern Africa

**DOI:** 10.1186/s13071-024-06394-3

**Published:** 2024-08-28

**Authors:** Andeliza Smit, Fernando Mulandane, Martinet Labuschagne, Stephané Heike Wójick, Choolwe Malabwa, Gourgelia Sili, Stephen Mandara, Zinathi Dlamkile, Rebecca Ackermann, Hannah Rose Vineer, Wilhelm Heinrich Stoltsz, Karine Huber, Ivan Gerard Horak, Darshana Morar-Leather, Benjamin Lawrence Makepeace, Luis Neves

**Affiliations:** 1 Department of Veterinary Tropical Diseases, Faculty of Veterinary Science, University of Pretoria, Gauteng, Republic of South Africa; 2 Biotechnology Center, Eduardo Mondlane University, Maputo, Mozambique; 3 Department of Genetics, Faculty of Natural and Agricultural Sciences, University of Pretoria, Gauteng, Republic of South Africa; 4 Central Veterinary Research Institute, Lusaka, Zambia; 5 Department of Basic Science, Faculty of Veterinary Medicine, University Jose Eduardo Dos Santos, Huambo, Angola; 6 Department of Animal Production Sciences, Marondera University of Agricultural Sciences and Technology, Marondera, Zimbabwe; 7 Department of Infection Biology and Microbiomes, Institute of Infection, Veterinary and Ecological Sciences, University of Liverpool, Liverpool, UK; 8 ASTRE, Univ Montpellier, CIRAD, INRAE, Montpellier, France

**Keywords:** Tick diversity, Phylogenetic, *Amblyomma*, Southern Africa, Systematics

## Abstract

**Background:**

*Amblyomma* spp. ticks, known for their long mouthparts, bright ornate appearance and aggressive hunting behaviour, are vectors of a number of important pathogens. In southern Africa, 17 *Amblyomma* spp. are currently documented. Of these species, *Amblyomma hebraeum* and *Amblyomma variegatum* have been well studied due to their wide geographical range and their status as competent vectors of pathogens that are of veterinary and medical importance. Studies on other *Amblyomma* spp. in southern Africa have been neglected, fostering ongoing debates on the validity of certain species such as *Amblyomma pomposum*. This study investigated the inter- and intra-species variation of *Amblyomma* ticks collected in southern Africa, focusing on resolving the dispute about *A. pomposum* and *A. variegatum* being distinct species.

**Methods:**

Four *Amblyomma* tick species were collected from Angola, Mozambique, South Africa, Zambia and Zimbabwe, and were identified morphologically as *Amblyomma eburneum* (208), *A. hebraeum* (4758), *A. pomposum* (191) and *A. variegatum* (2577) using identification keys. Gene amplification targeting the 12S and 16S rRNA, cytochrome oxidase I, cytochrome B and internal transcribed spacer-2 genes was conducted for 204 ticks, for which varying success was achieved during amplification for each of the markers. Maximum likelihood analyses were performed in IQ-TREE.

**Results:**

The phylogenetic topologies and ABGD analyses of each individual gene clustered *A. pomposum* within the *A. variegatum* clade, while clearly separating *A. eburneum* and *A. hebraeum* from all other species. None of the genetic markers indicated intraspecific structuring on the basis of geographical origin, despite great distances between sampling sites.

**Conclusion:**

Our study concludes that there is insufficient molecular evidence to differentiate *A. pomposum* and *A. variegatum *from each other. We highlight the need for whole mitochondrial genome sequencing of these two species to resolve the ongoing controversies. Furthermore, we propose mating and hybrid viability studies between the two species to confirm their reproductive isolation.

**Graphical Abstract:**

**Figure:**
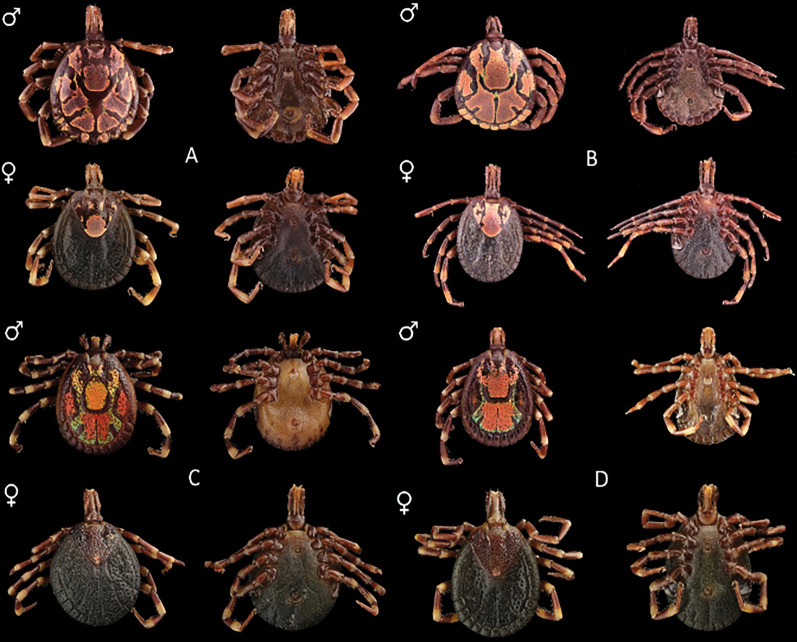

**Supplementary Information:**

The online version contains supplementary material available at 10.1186/s13071-024-06394-3.

## Background

The order Ixodida is divided into three families: Argasidae (also referred to as soft ticks), Ixodidae (also referred to as hard ticks) and Nuttalliellidae [1]. The most recent records collating valid tick names estimate 750 ixodid and 218 argasid species, as well as *Nuttalliella namaqua*, the only tick in the Nuttalliellidae family, and Deinocrotonidae (monotypic, but extinct) [2–4]. Of the 970 recognised species, 206 ixodids, 40 argasids and *N*. *namaqua* occur in the Afrotropical region of the world [2]. *Amblyomma*, one of the largest genera within the Ixodidae, is found on every continent except Antarctica, and is of major veterinary and public health concern in the Afrotropical region.

**Fig. 1:**
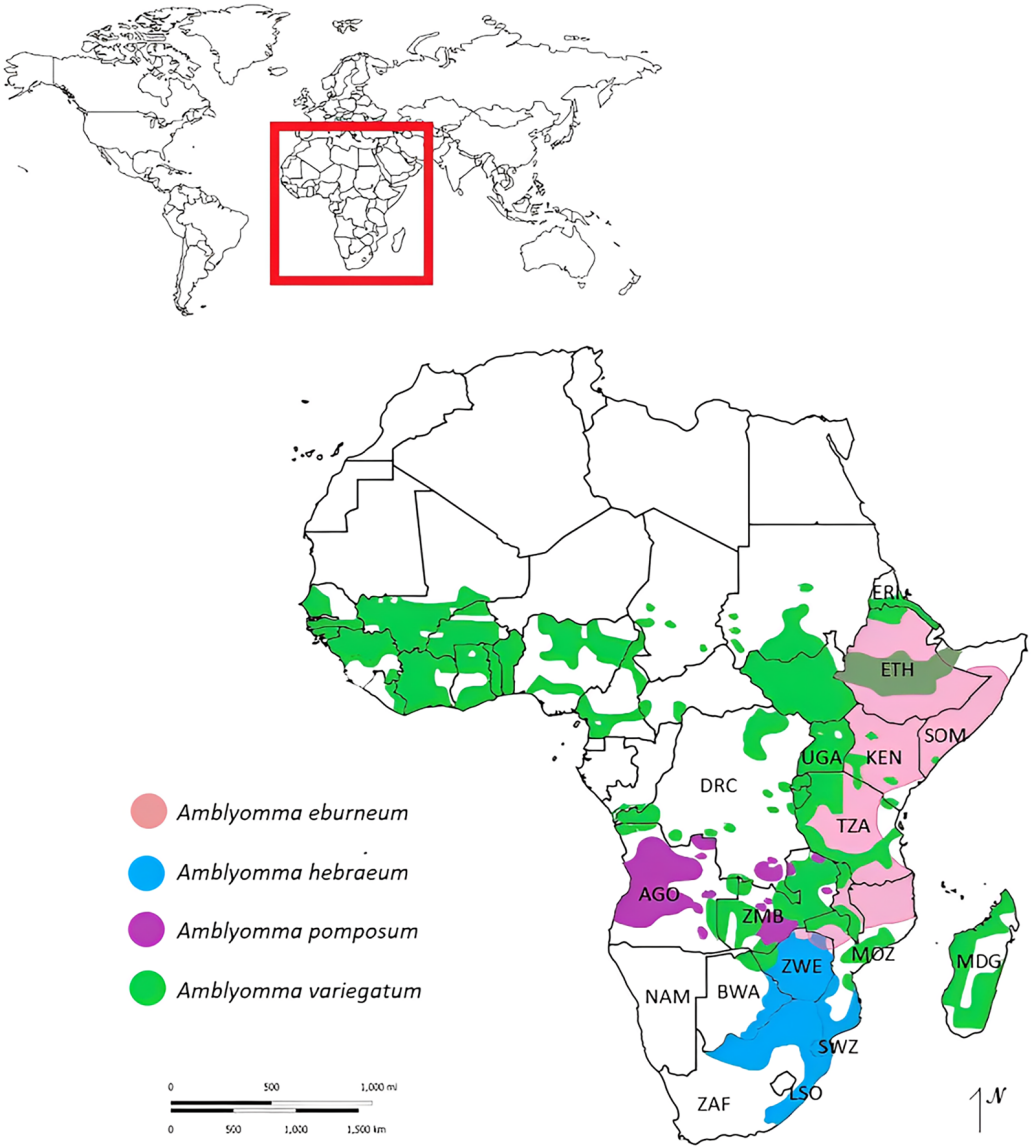
Map of Africa showing the historic distributions of *A*. *eburneum* (pink), *A*. *hebraeum* (blue), *A*. *pomposum* (purple) and *A*. *variegatum* (green) on the continent. Blank African map image was obtained from https://worldmapblank.com/blank-map-of-africa/ and modified with distribution information from Walker et al. [38] and Voltzit and Keirans [8]

*Amblyomma* spp. are known to be aggressive hunters with a vibrant and ornate appearance. The majority of the *Amblyomma* spp. that have been studied are known to be potential vectors of zoonotic pathogens such as *Rickettsia* spp.; however, they do not commonly feed on humans. They are vectors of several pathogens of veterinary importance including, but not limited to, *Ehrlichia ruminantium*, *Theileria mutans* and *Theileria velifera* [5, 6]. In the south-eastern parts of Africa, 21 *Amblyomma* species have been documented [6–8]. *Amblyomma variegatum* and *Amblyomma hebraeum* are the most predominant and widespread of the species, while distribution records of *Amblyomma pomposum* and *Amblyomma eburneum* are scant (Fig. 1).

Tick taxonomy in southern Africa dates back to 1778 and is focused on the morphological characteristics of adults, geographical distribution and host preference [9–14]. The first phylogenetic representation of ticks was depicted by Hoogstraal and Aeschlimann [15], but this representation has been revised over the years with increasing information obtained from molecular studies [16, 17]. Several studies have been conducted on the molecular systematics of *Amblyomma* spp.; however, most have included few or no *Amblyomma* spp. from southern African regions [18–21]. This limited representation of *Amblyomma* spp. from southern Africa has allowed for several incongruencies to have gone unanswered for decades.

The validity of certain *Amblyomma* spp. classifications has been placed under scrutiny. One noted controversy in southern Africa encompasses the *A. variegatum* group as described in Dias [22] and highlighted by Theiler and Salisbury [23] and Walker and Olwage [24]. *Amblyomma variegatum*, as described by Fabricius [25], is the oldest known species in the *A*. *variegatum* group, with a documented geographical spread as illustrated in Fig. 1. Dönitz [26] described *A*. *pomposum*, and although it is similar to *A*. *variegatum*, sufficient distinguishing phenotypic features are present to separate the two species morphologically. The main distinguishing features included coarse punctation on the conscutum and the fused central and cervical patches.

Robinson [27] described two male specimens collected in southern Rhodesia (now Zimbabwe) as *A*. *variegatum* var. *nocens*. Its distribution was designated to span from Makoni, Umtali (now Mutare) and Melsetter in Zimbabwe to Manica province in Mozambique and is found in bushveld ecoregions, at elevations of 2000–3000 feet [23, 27]. He noted that the main morphological difference between *A*. *variegatum* and *A*. *variegatum* var. *nocens* was that *A*. *variegatum* var. *nocens* had coarser punctations and was more vibrant in colouration. Robinson concluded in 1926 that the species he named *A*. *variegatum* var. *nocens* was synonymous with *A*. *pomposum* [26], which was given preference [22]. Theiler and Salisbury [23] examined and compared the two males, which Robinson [27] identified as *A. variegatum* var. *nocens* with *A. pomposum* samples and found that the morphologies did not resemble each other closely enough. Later, Dias described a new species, *A. variegatum* var. *govurensis* in Mozambique and compared its morphology with that of *A*. *variegatum* and *A*. *pomposum*. He described *A. variegatum* var. *govurensis* as distinguishable from the type specimens of *A*. *variegatum* and *A*. *pomposum* by large and coarse punctations, while the patterns were more vibrant and intense. Dias [22] attempted to revise the *A. variegatum* group, to include his newly described species *A. variegatum* var. *govurensis*; however, upon examination of the samples from Robinson [27], Dias concluded that his *A. variegatum* var. *govurensis* was identical to *A*. *variegatum* var. *nocens* and thus synonymous with *A*. *pomposum*.

During Dias’s revision of the *A*. *variegatum* group, he went on to note that the records of *A*. *pomposum* in eastern Africa as described by Dönitz [26] morphologically resembled the specimens that they found in Mozambique; however, the *A*. *pomposum* from western and central Africa did not match the description of the species by Dönitz [26]. Dias [22] proposed a new species name, *Amblyomma superbum*, which he illustrated in Dias [28], for the *Amblyomma* spp. in central and western Africa. This new nomenclature was rejected [23], and “*A*. *pomposum*” is still used to describe the species occurring in the central and western parts of Africa [23]. In light of these morphological debates, Theiler and Salisbury [23] examined all the reference material they had access to and advocated for the re-establishment of *A*. *variegatum* var. *nocens* as *Amblyomma nocens* Robertson 1911, with a geographic distribution range confined to latitudes 18–22°S. This change in nomenclature was also rejected [29].

To date, there is still disagreement regarding the diversity of *Amblyomma* spp. in Mozambique. It is unclear whether these morphological variations in the *Amblyomma* spp. found in Mozambique are due to the ticks being different species, or subspecies, or merely due to intraspecific heterogeneity. Although this disagreement on the geographical distribution of *A. variegatum* and *A. pomposum* in Mozambique has been ongoing for decades [8, 22–24], no molecular evidence has been provided to help resolve this question.

Molecular studies on *Amblyomma* spp. in Africa have been limited to date, focusing on mitochondrial genetic markers, such as the cytochrome oxidase subunit 1 (*coi*) gene, 12S rRNA and 16S rRNA genes, and nuclear markers, such as 18S rRNA and 28S rRNA [18, 30, 31]. Phylogenetic analyses with the use of these markers showed varying differentiating capabilities. To increase phylogenetic resolution, whole mitochondrial analyses have been conducted and proven to be highly effective in distinguishing between closely related species [16, 21, 32, 33]. To date, whole mitochondrial genome (mitogenome) sequencing is still considered expensive, limiting the number of mitogenomes available on sequence repositories and limiting its use in developing countries. Concatenation of several molecular markers into a “super-matrix” is a commonly used tool to differentiate between species, although debates on its accuracy has been long-standing [34–36].

Thus, this study investigated the intra- and inter-species variation of *A. eburneum*, *A. hebraeum*, *A. pomposum* and *A. variegatum* ticks collected in southern Africa using molecular techniques (concatenation of four mitochondrial markers), with the aim to resolve the taxonomic controversy between *A*. *pomposum* and *A*. *variegatum* in southern Africa.

## Methods

### Sample collection

**Fig. 2:**
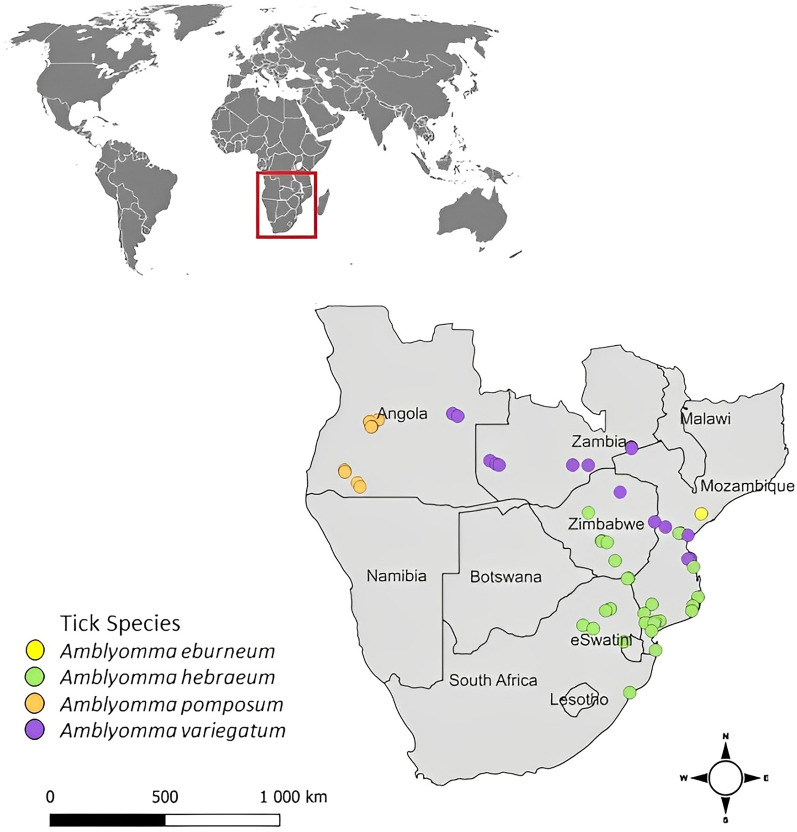
Map of southern Africa, illustrating the collection points during the 2020–2022 sampling period of *Amblyomma* spp. The colour of the dot represents the species of *Amblyomma* tick collected at the sampling point

*Amblyomma* spp. ticks were collected in Angola, Zambia and Zimbabwe (Fig. 2) by means of hand collections from cattle and goats. In Mozambique, *Amblyomma* spp. ticks were also collected from livestock, and legally hunted wildlife (Fig. 2; Additional file 1: Text S1). Animals were visually inspected for tick infestations before collections were made. Due to the bright ornate patterning in *Amblyomma* spp., these ticks can be spotted from a distance. This prevented the unnecessary restriction and sampling of animals that would not provide a sufficient number of ticks. Once an infested animal was spotted and restrained, complete tick collections were done by hand from the animal to ensure optimal species representation. Tick samples were taken from the whole body of the animal, paying particular attention to the predilection sites such as the dewlap, perineum, axilla, udder, or interdigital spaces. Collections were performed during the optimal season for adult ticks in each country: in Angola during March 2022, in Mozambique from February 2021 to April 2021 and again in November 2021, in Zambia during April 2022 and July 2022 and in Zimbabwe from March 2022 to June 2022. *Amblyomma* spp. ticks from South Africa were obtained from Dlamkile et al. [37] (Additional file 1: Text S1). Collected ticks were stored in 70% ethanol and transported to the Department of Veterinary Tropical Diseases, University of Pretoria, Onderstepoort. The ticks were morphologically identified to the species level, using identification keys obtained from Walker et al. [38] and Voltzit and Keirans [8]. Morphological characteristics were documented using the Fujifilm XT-4 camera, and images were stacked using Capture One Pro 9 and Zerene Stacker software version 1.04 [39, 40].

### DNA extraction and amplification

DNA from all ticks were individually extracted using the Chelex 100 resin method (total final volume of 100 µl) as described by Smit et al. [41] and stored at –20 °C for downstream use. Molecular characterisation was conducted targeting four mitochondrial (12S rRNA gene [42], 16S rRNA gene [43], cytochrome B (*cytB*) [44] and cytochrome oxidase subunit 1 (*coi*) [45]) genes and the nuclear internal transcribed spacer 2 (*ITS2*) region [20]. These markers were selected on the basis of reference sequence availability on GenBank and as the most popular loci used in the literature. Four male and four female ticks per species per region, if available, were selected for DNA extraction. In total 204 ticks were used: *A*. *eburneum* (*n* = 13), *A*. *hebraeum* (*n* = 92), *A*. *pomposum* (*n* = 26) and *A*. *variegatum* (*n* = 73). Amplification master-mixes are described below. Primer, master-mix and annealing temperatures can be located in Additional file 2: Dataset S1. The general polymerase chain reaction (PCR) cycling conditions for each marker comprised an initial denaturation at 98 °C for 10 s, followed by 10 cycles of denaturation at 98 °C for 1 s, annealing at *x* °C for 5 s and extension at 72 °C for 15 s. This was followed by 30 cycles of amplification with denaturation at 98 °C for 1 s, annealing at *y* °C for 5 s and extension at 72 °C for 15 s. Final extension was performed at 72 °C for 15 s.

The PCR products were separated on a 1.5% agarose gel and visualised using the Bio-Rad gel documentation system with assisting visualisation programming. All samples that had visible bands were sent to the Central Analytical Facility (CAF), Stellenbosch, South Africa, for Sanger sequencing in both directions.

### Phylogenetic analysis

Each gene was analysed separately. Sequences were manually corrected on CLC main workbench version 23.0.2 (developed by CLC Bio, http://www.clcbio.com), and compared with those available in the GenBank database using the Basic Local Alignment Search Tool (BLAST; https://www.ncbi.nlm.nih.gov/genbank/) [46]. Reference sequences were obtained from GenBank and used for comparison purposes (Additional file 3: Dataset S2). All reference sequences that were available of *A*. *eburneum* and *A*. *pomposum*, as well as for *cytB*, were used due to their limited number. *Amblyomma hebraeum* and *A*. *variegatum* had a larger variety of available sequences; therefore, several sequences were selected that were of similar length to the sequences generated in the study. The outgroup was selected to be *Amblyomma americanum*, as it is sufficiently distant from the species obtained in this study to serve as a well-defined root. Contigs were assembled, sequence orientations were confirmed and alignments were generated alongside reference sequences using the online MAFFT version 7 (developed by http://mafft.cbrc.jp/alignment/server/index.html) with default parameters. The frameshift and stop codon detection was conducted for each matrix using MACSE version 2.07 using the invertebrate mitochondrial code [47]. The aligned matrices were manually viewed, edited and truncated using MEGA 11 [48]. The best fit model was determined for each individual gene using ModelFinder [49] on the IQ-TREE version 1 software [50]. Maximum likelihood (ML) analyses were conducted on IQ-TREE version 1 using 1000 bootstrap replicates. The resulting trees were visualised and edited in iTOL version 6.8 [51]. Sequences that were identical to each other were removed from the trees to increase readability. The evaluation criteria for the bootstrap values were as follows: values of 75% and lower were considered nonsignificant, between 75 and 94% were considered moderately significant and values of 95% and higher represented high statistical support [52].

A concatenated file was constructed including *12S*, *16S*, *coi* and *cytB* genes. Alignments for each of the individual genes were done on MAFFT. The aligned sequences were then concatenated into a super-matrix using MEGA 11. Model selection and ML analysis was done using IQ-TREE. Partitioning was applied to the super-matrix [53] with a 1000 bootstrap replicates using the ultrafast bootstrapping method [54]. The evaluation criteria for the ultrafast bootstrap values were as follows: values of 95% and higher represented high statistical support, while values lower than 95% were considered unsupported [55].

### Barcode gap analysis

Groups based on *Amblyomma* spp. were created in Mega version 11, and the intra- and inter- species variation of these groups were compared. A pairwise distance matrix (p-distance) was obtained for 12S rRNA and 16S rRNA genes, *coi*, *cytB* and the concatenated alignment using default parameters with 1000 bootstrap replicates to calculate the standard error (SE). Barcode gap analysis was conducted using the Automatic Barcode Gap Discovery (ABGD) tool on the online web server (https://bioinfo.mnhn.fr/abi/public/abgd/abgdweb.html) [56]. Analysis was conducted using default parameters (*P*_min_ = 0.001; *P*_max_ = 0.1; 10 steps and relative gap width *X* = 1.5) using the Kimura (K80) TS/TV model.

## Results

**Fig. 3:**
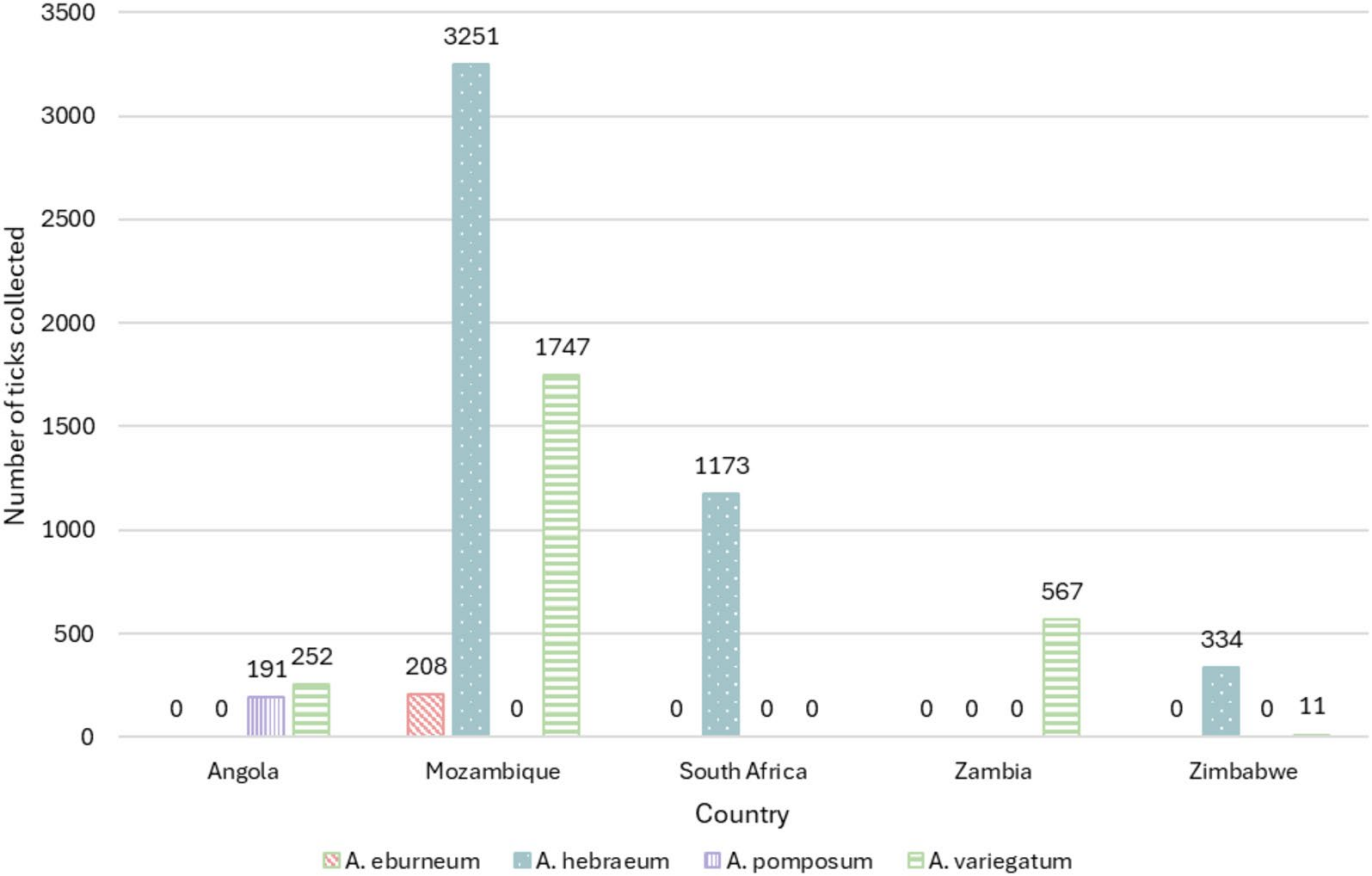
The number and species of *Amblyomma* ticks collected from Angola, Mozambique, South Africa, Zambia and Zimbabwe over the course of this study

**Fig. 4:**
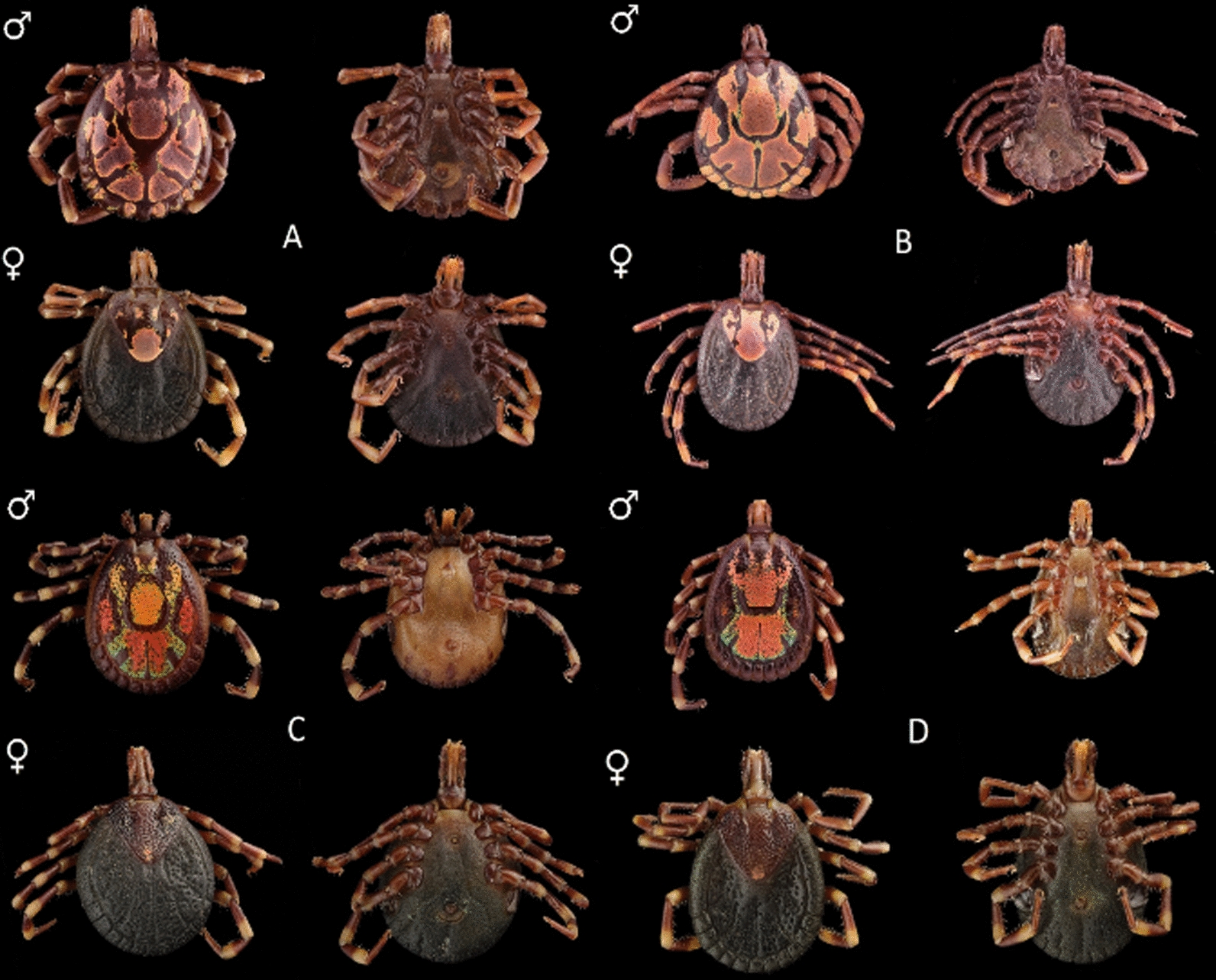
Morphological comparison of males (indicated by ♂) and females (indicated by ♀), where plate **A** represents *A*. *eburneum*, plate **B** represents *A*. *hebraeum*, plate **C** represents *A*. *pomposum* and plate **D** represents *A*. *variegatum*. Dorsal and ventral views are illustrated. Photographs were taken by Prof. Melvyn Quan and Ms. Zandile Mkhize

In total, 7734 adult *Amblyomma* spp. ticks were obtained from Angola, South Africa, Mozambique, Zambia and Zimbabwe (Fig. 3). These ticks were identified morphologically to the species level; representative specimens of each sex are illustrated in Fig. 4. Four species were observed [*A. eburneum* (*n* = 208), *A. hebraeum* (*n* = 4758), *A. pomposum* (*n* = 191) and *A. variegatum* (*n* = 2577)], and the main morphological differences are summarised in Table 1. *Amblyomma eburneum* was only collected in one location in central Mozambique (18°S; Fig. 2). *Amblyomma hebraeum* was collected in the northeastern parts of South Africa, in the southern and central regions in Zimbabwe and in the southern parts of Mozambique (below 21°S), with two sampling points extending beyond what is currently documented as the *A*. *hebraeum* endemic zone (19°S; Fig. 2). *Amblyomma pomposum* was only collected in Angola in the central regions expanding westwards (Fig. 2). *Amblyomma variegatum* was collected in central northern regions of Mozambique (above 21°S), in northern regions of Zimbabwe, latitudinally across Zambia and in eastern parts of Angola (Fig. 2).

**Table 1:** Main morphological differences between *Amblyomma eburneum*, *Amblyomma hebraeum*, *Amblyomma pomposum* and *Amblyomma variegatum*

	*Amblyomma eburneum* (Fig. 4A)	*Amblyomma hebraeum *(Fig. 4B)	*Amblyomma pomposum *(Fig. 4C)	*Amblyomma variegatum *(Fig. 4D)
*Males*				
Colouration	Red-brown with beige patterning	Red-brown with metallic beige patterning	Black-brown with red, orange and green patterning	Black-brown with red, orange and green patterning
Vibrance	Vibrant and metallic	Vibrant and metallic	Vibrant and metallic	Muted and metallic
Eyes	Slightly convex	Flat	Distinctly convex	Distinctly convex
Punctations	Small and shallow	Small and shallow	Large and densely scattered	Medium or small and densely scattered
Mesial patch	Square, indistinctly connected to posterior enamel areas	Elongated, reaching the capitulum	Oval, unconnected to other enamelled patches	Square, distinctly connected to falciform stripe
Falciform stripe	C-shaped	Triangular shaped	Claw shaped	Horn-like appearance
Posterior medial stripe	Straight and wide	Straight and narrow	Straight and narrow	Extremely narrow
Lateral patches	Ovoid-shaped, distinctly present	Complex shape, distinctly present	Large trapezoid distinctly present	Generally absent (if present they are indistinct and small)
Festoon colouration	10/11 (central festoon is uncoloured)	9/11 (two outer festoons are uncoloured)	0/11	0/11
*Females*				
Punctuation	Small	Deep near eyes; shallow central and scapular areas	Large and densely scattered	Small or medium, densely scattered
Mesial patch	Large circular patch with two small central patched	Large patch covering whole central third	Small, circular and faint	Small, circular and clear
Lateral patch	Two small unconnected circular patches	Large c-shaped	Indistinct but present	Indistinct but present
Posterior angle	Broad	Broad	Narrower	Broad

### Phylogenetics

From the selected 204 *Amblyomma* ticks used for phylogenetic analyses, 24 (11.8%) did not amplify with any of the selected markers and were removed from further analyses. The remaining 180 samples had varying success with amplification and contig construction amongst the genes; success rates were 69.4% (125/180) for 12S rRNA, 57.2% (103/180) for 16S rRNA, 71.1% (128/180) for *coi*, 64.4% (116/180) for *cytB* and 69.4% (125/180) for ITS2. Although the majority of the sequences amplified using ITS2, it was excluded from further analyses due to the genetic variability only consisting of uninformative single nucleotide mutations preventing any systematic analyses. Difficulties arose during the contig construction of the 16S rRNA sequences due to low sequence quality. In contrast, 12S rRNA and *coi* were the most successful in sample amplification and produced sequences of good quality resulting in a high rate of contig assembly of these sequences. The number of sequences per species analysed for each gene varied (Table 2). All sequences were deposited in GenBank (Additional file 3: Dataset S2).

**Table 2:** The number of sequences analysed for the pairwise distance matrix and for the Automatic Barcode Gap Discovery (ABGD) analysis

Species/number of sequences analysed	*12S*	*16S*	*coi*	*cytB*	Concatenated
*Amblyomma eburneum*	12	12	15	12	11
*Amblyomma hebraeum*	77	50	82	56	30
*Amblyomma pomposum*	11	16	5	19	2
*Amblyomma variegatum*	42	32	38	33	7
Outgroup (*Amblyomma americanum*)	1	1	1	1	1
Total	143	111	141	121	51

The 12S rRNA gene exhibited an average amplified fragment length of 400 bp, while the final fragment length for the alignment was 383 bp. The 16S rRNA had the shortest average amplified fragment length at 340 bp, with the final fragment alignment length for the 16S rRNA alignment was 328 bp. The average length of the amplified fragment of *coi* was bp, with the final fragment length in the *coi* alignment being 650 bp. Lastly, the average amplified fragment length for *cytB* was 500 bp, while the final fragment length for the *cytB* alignment was 483 bp.

The average intraspecific pairwise distances and the average interspecific pairwise distances are given in Additional file 4: Dataset S3. In nearly all interspecific evaluations, *A. pomposum* and *A. variegatum* had the least amount of distance between them. The intraspecific pairwise distances were on average lower than the average interspecific pairwise distances. The pairwise difference between individual sequences for each gene were also compared (Additional file 5: Dataset S4 to Additional file 9: Dataset S8).

When evaluating the 12S rRNA estimations of evolutionary divergence between each of the sequences, low levels of intraspecific variation were observed. The intraspecific variation for *A*. *eburneum* was the same as the interspecific variation between *A*. *eburneum* and *A*. *hebraeum*. *Amblyomma hebraeum* had low intraspecific variation reflecting high levels of co-linearity. The interspecific variation between *A*. *pomposum* and *A*. *variegatum* was nearly identical to the intraspecific variation of *A*. *variegatum*.

The ML phylogenetic topology of 12S rRNA depicted a clear separation between *A*. *hebraeum* from all other species, forming a single clade. This separation was corroborated by the ABGD analysis, in which *A*. *hebraeum* clustered in a single operational taxonomic unit (OTU). The *A*. *eburneum* sequences were observed to be divided into three main clades, separated from all the other species. The first clade (OTU 3) was separated from the *A*. *variegatum/pomposum* cluster, forming a sister clade with the remaining *A*. *eburneum* and *A*. *hebraeum* sequences with high bootstrap support, but contained only two sequences. The main clade of *A*. *eburneum* sequences (OTU 4) was resolved with strong support, while a third sequence (OTU 2) branched from this main group with moderate support.

The *A*. *pomposum* and *A*. *variegatum* sequences formed two main clades. The first was a minor clade containing only five *A. variegatum* sequences, all originating from Mozambique. The second, much larger clade exhibited strong support (100 bootstrap value) for a subclade consisting of nine sequences (four from Zambia and five reference sequences from GenBank). Otherwise, phylogenetic evidence for population structure within the larger clade was poor, including for *A. pomposum* as a distinct subclade. In the ABGD analysis, the *A*. *pomposum* and *A*. *variegatum* sequences were divided into three OTUs (#5, #7 and #8). While *A*. *pomposum* sequences segregated together in the tree, they were grouped with a majority of *A*. *variegatum* sequences into a single OTU (#5). Notably, the minor clade from Mozambique and the subclade described above of nine Zambian and published reference sequences formed a separate OTU (#8). A final, singleton OTU (#7) consisted of an *A*. *pomposum* sequence located on a long branch, although this was not well supported in the tree. This sequence contained several single nucleotide polymorphisms (SNPs) when compared with others of the same species (Additional file 10: Fig. S1).

The 16S rRNA pairwise sequence matrix revealed low intraspecific variation in both *A*. *hebraeum* and *A*. *pomposum*, while interspecific variation was lower between *A*. *pomposum* and *A*. *variegatum* as compared with other species. The ML topology of the 16S rRNA depicted a clear separation between *A*. *eburneum*, *A*. *hebraeum* and the *A*. *variegatum* complex. The *A*. *hebraeum* sequences formed two distinct clades, sister to an *A*. *eburneum* clade (OTU 4), with high bootstrap support. However, *A. hebraeum* was classified into a single OTU (#5) in the ABGD analysis. The *A*. *eburneum* specimens were divided into two main clades, one sister to *A*. *hebraeum* and the other separated from all other species (OTU 2). The larger clade (OTU 2) contained nine sequences from this study alongside the only reference sequence from GenBank, whereas the other cluster (OTU 4) comprised only two sequences from this study.

The *A*. *variegatum* and *A*. *pomposum* cluster was divided into two clades. The first clade consisted of a single *A*. *variegatum* reference sequence from Sao Tome and Principe, which was supported with a moderate bootstrap value. The second clade comprised the remaining *A*. *variegatum* and *A*. *pomposum* sequences, and these were poorly resolved, with a lack of phylogenetic support for population structuring. While most *A*. *pomposum* sequences were located together in the tree, one reference sequence from Zambia was nestled among *A*. *variegatum*. Regardless, there was no bootstrap support for the separation of *A*. *pomposum* from *A*. *variegatum*, and this was corroborated by the ABGD analysis, which defined a single OTU (#3) encompassing all *A*. *pomposum* and *A*. *variegatum* sequences (Additional file 11: Fig. S2).

The analysis of the *coi* pairwise sequence matrix illustrated higher intraspecific variation compared with the other markers, except for *A*. *hebraeum*, which still exhibited low levels of polymorphism. The ML phylogenetic topology for *coi* depicted a clear separation of *A. hebraeum* from the other *Amblyomma* species. Although *A*. *hebraeum* was divided into two main clades, supported by a moderate bootstrap value, it formed one OTU (#5) in the ABGD analysis. The first clade consisted of two *A*. *hebraeum* sequences from Zimbabwe, while the second clade consisted of all other remaining sequences generated during this study alongside reference sequences from GenBank. Similarly, *Amblyomma eburneum* was divided into two well-supported clades. The first contained only two sequences obtained from this study (OTU 3), whereas the other (OTU 2), a sister clade to *A*. *hebraeum*, consisted of the bulk of the sequences from this study alongside the two reference sequences currently available on GenBank. *Amblyomma pomposum* and *A*. *variegatum* were segregated into a single clade with no bootstrap support for their separation. Within *A*. *variegatum*, 14 sequences 7 from Zambia, four from Mozambique, 1 from Senegal and 1 from Guinea-Bissau) formed a well-supported subclade, although they were not classified as a separate OTU. Moreover, within the main *A*. *pomposum/A. variegatum* clade, one *A*. *pomposum* sequence (AHB015) branched from the others with high bootstrap support. Nevertheless, the ABGD analysis grouped *A*. *pomposum* and *A*. *variegatum* into a single OTU (#4) (Additional file 12: Fig. S3).

The *cytB* pairwise distance matrix depicted low levels of intraspecific variation, especially for *A*. *eburneum* and *A*. *hebraeum*. The interspecific variation between *A*. *pomposum* and *A*. *variegatum* was much lower than between the other species. The *cytB* ML phylogenetic topology depict a clear separation between *A*. *eburneum* and *A*. *hebraeum* with moderate bootstrap support, and these sister clades were separated into their own OTUs (#2 and #3). Both *A*. *hebraeum* and *A*. *eburneum* exhibited evidence of intraspecific population structure, with some branches displaying moderate or strong support. The *A*. *variegatum* sequences were divided into two main subclades, with high bootstrap support. The first subclade contained representatives from Angola, Mozambique, Zambia and Zimbabwe, while the second consisted of *A*. *pomposum* sequences alongside some *A*. *variegatum* specimens from Mozambique and Zambia. These two subclades were grouped into a single OTU (#4) by ABGD analysis, with the exception of one *A. variegatum* sequence from Mozambique that formed its own OTU (#5) on a long branch. When examining this sequence, several SNPs were evident (Additional file 13: Fig. S4).

**Fig. 5:**
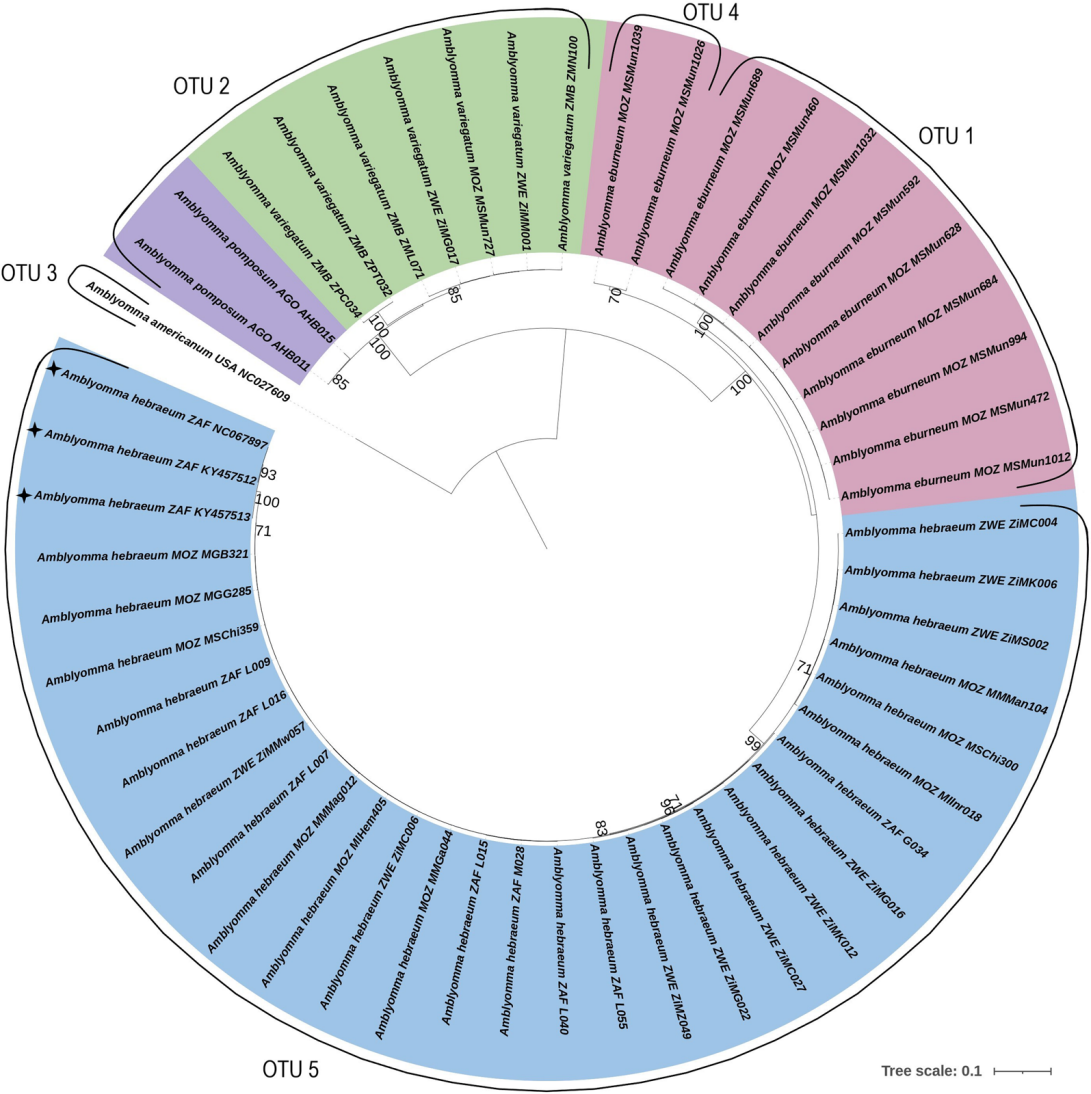
Maximum likelihood (ML) analysis of all concatenated genes. The ML analysis used the individual models for each gene: HKY + F + G4 for 12S rRNA, K3Pu + F + I for 16S rRNA and HKY + F + I for both *coi* and *cytB*. Bootstrap support is indicated at each branch node, and values under 70 were removed. Blue, pink, green and purple highlighted regions indicate *A*. *hebraeum*, *A*. *eburneum*, *A*. *variegatum* and *A*. *pomposum*, respectively. Species names are included with a three-letter country code and the sample name or GenBank accession number. Country codes: AGO, Angola; MOZ, Mozambique; ZAF, South Africa; ZM, Zambia; and ZWE, Zimbabwe.GenBank reference strains are marked with ✦

For the concatenated alignment (12S rRNA and 16S rRNA genes, *coi* and *cytB*), the pairwise distance matrix illustrated low levels of intraspecific variation, especially for *A*. *hebraeum*. The interspecific variation between *A*. *pomposum* and *A*. *variegatum* was nearly five times lower than that between the other species. The ML phylogenetic topology indicated clear separation between all four species with high bootstrap support (Fig. 5). Within the *A*. *hebraeum* clade, multiple branches were observed with moderate to high bootstrap support; however, only one OTU (#5) was indicated by ABGD analysis. *Amblyomma eburneum* was separated into two clades with moderate to high bootstrap support. The smaller clade contained the two sequences from Mozambique that clustered separately in the individual gene trees, whereas the larger clade comprised the remaining *A*. *eburneum* sequences. This separation was supported by the ABGD analysis, which divided *A*. *eburneum* into two OTUs (#1 and #4). Unfortunately, only two *A*. *pomposum* sequences could be used in the concatenated analysis due to low amplification and sequencing success as described above. These two sequences were separated from *A*. *variegatum* with high bootstrap support, but the ABGD analysis placed both species in the same OTU (#2).

## Discussion

This study investigated the intra- and inter-species variation of *Amblyomma* spp. collected in southern Africa. In total 7734 adult *Amblyomma* ticks were collected and morphologically identified as *A*. *eburneum*, *A*, *hebraeum*, *A*. *pomposum* and *A*. *variegatum*. However, on the basis of our analyses of multiple genes, we cannot conclude that *A*. *pomposum* and *A*. *variegatum* are distinct species.

*Amblyomma eburneum* was only collected in one location in central Mozambique (18°S) from wildlife (mainly African buffalo, *Syncerus caffer*). Information on *A*. *eburneum* is scant; however, it is described as an East African species with a documented geographical distribution as illustrated in Fig. 1 [8, 41]. The sympatric occurrence between *A*. *eburneum* and *A*. *variegatum*, as found in Mozambique, is uncommon for the genus *Amblyomma* [57]. While no definitive conclusions can be drawn for this co-occurrence, lack of sexual interference between the two species is one hypothesis. This sympatric distribution between *A*. *eburneum* and *A*. *variegatum* [41] and the parapatric distribution between *A*. *hebraeum* and *A. variegatum* [57] would be an ideal model for further investigation into the sexual behaviours of these tick species and their impact on interspecific competition and boundary formation.

*Amblyomma hebraeum* was collected in northeastern parts of South Africa, southern and central regions of Zimbabwe and in southern areas of Mozambique (below 21 °S), which agrees with the current literature [7, 24, 38, 58]. *Amblyomma hebraeum* was also collected at two sampling points extending beyond what is currently documented as its endemic zone (19°S), indicating expansion of its geographical distribution. Indeed, it has been documented that the geographical distribution of *Amblyomma* spp. has widened in the past few years [59]. The main culprit for these expansions is always assumed to be climate change; however, albeit a major factor, it is not the only cause for these observations [59]. The transportation of livestock within and across countries greatly impacts the distribution potential of ticks. Regarding our data, it is more likely that *A*. *hebraeum* was introduced from other parts of Mozambique or from South Africa by the transportation of infested animals to the new region; this is supported by the fact that the parapatric boundary between *A*. *hebraeum* and *A*. *variegatum* in Mozambique has been maintained for almost a century, with no geographical expansion [57]. Both male and female *A*. *hebraeum* ticks were collected, which could lead to the establishment of this species in this new geographical area. It would be of interest to continuously observe this subpopulation of *A*. *hebraeum* in an area where *A*. *variegatum* is known to occur and document the competitive behaviour between the two species.

The geographical spread of *A*. *pomposum*, as illustrated in Fig. 1, was described by Robinson [60], Walker and Olwage [24] and Petney et al. [58] to range from Angola to the western regions of Zambia, and northwards to the southern parts of the Democratic Republic of Congo. In the present study, *Amblyomma* spp. were also collected from the western parts of Angola and latitudinally to the eastern parts of Zambia. We found *A*. *pomposum* to be restricted to the central-western parts of Angola, whereas *Amblyomma* specimens collected in eastern Angola were identified as *A*. *variegatum*. The most probable reason for this discrepancy is the misidentification of *A*. *variegatum* as *A*. *pomposum* in these earlier studies due to their close morphological appearance. However, cattle are known to migrate from Angola to Zambia during drier periods when the marshlands are not flooding; thus, cattle harbouring *A*. *pomposum* could possibly be found in Zambia at these times. Yet, if this was the case, we would expect to see the establishment of *A*. *pomposum* in eastern Angola and Zambia, which was not observed in this study. Since no voucher material of *A*. *pomposum* from Zambia is available and the one of the 16S rRNA sequences for *A*. *pomposum* on GenBank appears to be an outlier in our analyses, we maintain that *A*. *pomposum* is unlikely to occur in Zambia. In this study, *A*. *variegatum* was collected in Angola, Mozambique, Zambia and Zimbabwe, corresponding with the records of Petney et al. [58] and Walker and Olwage [24].

The most predominant collected species was *A*. *hebraeum* from South Africa and Mozambique, while the species with the lowest representation was *A*. *pomposum* from Angola. The low recovery of *A*. *pomposum* and *A*. *variegatum* in Angola could be ascribed to the collections occurring in March, which is at the end of the season for the adult stage (the months when the maximum infestations occur have been documented as November and December [58]). *Amblyomma eburneum* was also collected in low numbers, but this can be attributed to the difficulty of collecting ticks from wildlife species, as we were highly dependent on the number of wild animals that was legally hunted in the timeframe of this study. The low prevalence of *Amblyomma* spp. from Zimbabwe was likely the result of a vigorous campaign by the government to assist livestock farmers in treating their cattle against ticks by encouraging regular dipping of the animals with acaricides, in an attempt to control major outbreaks of bovine theileriosis.

This study aimed to provide insight into the unresolved debate that has been ongoing for decades as described in the introduction. Both Robinson [27] and Dias [22, 28] described morphological variation in *A*. *variegatum* found in Mozambique and suggested the new species names *A. variegatum* var. nocens and *A. variegatum* var. *govurensis*, respectively. Dias [22] also examined what was described as *A*. *pomposum* from Angola and concluded that it does not resemble the *A*. *pomposum* as described by Dönitz [26], suggesting a new species description as *Amblyomma superbum*. In this study, morphological variation in the collected *A*. *variegatum* ticks were observed; however, the majority resembled that of the original description by Fabricius [25]. Morphology is an important aspect in tick taxonomy, highlighting the importance of variation in ornamentation. These conflicting observations of the morphology in *A*. *variegatum* is reminiscent of the controversy regarding the *Amblyomma marmoreum* complex [61].

The *Amblyomma marmoreum* complex encompasses five African species, namely: *A*. *marmoreum* sensu stricto, *A*. *sparsum*, *A*. *falsomarmoreum*, *A*. *nuttalli* and *A*. *paulopunctatum* [61]. As with this study, the *A*. *marmoreum* complex was placed under scrutiny due to a lack of genetic and ecological data on its members. Although meticulous descriptions of the five species in the *A*. *marmoreum* complex are available, identification remains a challenge, and misidentification often occurs, even confusing these species with other *Amblyomma* spp. outside of the complex [23, 42, 61]. Cotes-Perdomo et al. [61] was, however, able to differentiate between the species with the use of molecular techniques targeting a large part of the mitogenome. Overall, they were able to concatenate and compare 13 protein-coding genes and two ribosomal genes of several *Amblyomma* spp.

In an attempt to provide clarity on the phylogenetic positioning of *A*. *pomposum* and *A*. *variegatum*, molecular analyses were conducted on four *Amblyomma* spp. of southern Africa using 12S rRNA, 16S rRNA, *coi*, *cytB* and ITS2 molecular markers. The ITS2 marker was, however, excluded from further analyses due to uninformative single nucleotide mutations. Our research provides additional sequences that have been deposited in the GenBank database for all other markers utilised here, including the first for the 12S rRNA and *cytB* genes of *A*. *eburneum* and first for the 12S rRNA, *coi* and *cytB* genes of *A*. *pomposum*. During the course of this study, several challenges with amplification occurred for all genes. The Chelex extraction method has several drawbacks including the rapid degradation of the extracted DNA after 2 years of storage and the effects of long-term storage on the binding of impurities to DNA. Singh et al. [62] noted that samples extracted with the Chelex 100 resin method and stored for extended periods of time tended to contain contaminants such as proteins attached to the DNA helix, and these prevented successful PCR reactions. The importance of the impact of storage time on DNA integrity was evident through the successful amplification of *cytB*, which was conducted with DNA extracted less than 1 year previously, compared with the less successful amplification of 12S rRNA, 16S rRNA, *coi* and ITS2, which were conducted using DNA stored for approximately 2 years. Additionally, several amplified products did not generate sequences of good quality, and were thus excluded from further analysis, lowering the sample size. The *coi* gene marker was particularly difficult to amplify in *A*. *pomposum* samples, and those that did produce bands for these markers did not provide high-quality sequences. For future studies, it would be ideal to use recently extracted DNA when using the Chelex extraction method, and DNA should be stored at −80 °C, which would maintain the integrity of the DNA for a longer period, or alternative DNA extraction methods should be used that will not compromise the DNA integrity during storage.

The ML and ABGD analysis indicated that *A*. *eburneum* and *A*. *hebraeum* were sister groups but consistently formed their own OTUs. In the case of *A. hebraeum*, a single OTU was resolved in the single-gene and concatenated analyses with relatively low intraspecific variation. *Amblyomma eburneum* was divided into three OTUs in the 12S rRNA analysis; two OTUs in the 16S rRNA, *coi* and concatenated analyses; and a single OTU in the *cytB* analysis. In all the ML trees, excluding *cytB*, two *A*. *eburneum* samples from Mozambique (MSMun 1039 and MSMun 1026) were separated from the main clade with moderate to high bootstrap support, although it is unclear why this is the case. The samples were collected in the same area as all other *A*. *eburneum*, on the same hosts and over the same timeframe. A possible explanation for both the low polymorphism observed in *A*. *hebraeum* and the higher genetic diversity observed within *A*. *eburneum* is the influence of host dispersal on gene flow between generalist and specialist ectoparasites [63]. Matthee [63] documented that *A*. *hebraeum*, which occurs on hosts with low global range values (i.e. that are located close together), showed the lowest levels of population differentiation and high levels of gene flow. These findings contradicted those of other generalist species studied by Matthee [63]. *Amblyomma hebraeum* was also documented to have the lowest haplotype diversity amongst generalist species [63]. In contrast, *A*. *eburneum*, is a specialised species that occurs only on wild ungulates, and in Mozambique, is restricted to hosts with a limited geographical range. According to Matthee [63], the low gene flow could results in high population differentiation. We speculate that the two unique *A*. *eburneum* samples could be from a subpopulation that has undergone more genetic isolation compared with the main clade. This suggests an opportunity to study the intraspecific diversity of *A*. *eburneum* across its whole latitudinal range, from Ethiopia to Mozambique.

Strikingly, *A. pomposum* clustered with *A*. *variegatum* in the ABGD analyses for all the individual genes and in the concatenated analyses. Similarly, in the single-gene trees, *A*. *pomposum* samples were nested within the *A*. *variegatum* clade, usually as a single cluster with low bootstrap support. However, in the concatenated ML tree, separation of *A. pomposum* from *A. variegatum* was well supported, albeit at a short genetic distance and with limited sequences available for *A*. *pomposum*. *Amblyomma variegatum* formed a single OTU in all analyses except for the 12S rRNA gene and *cytB*. In the 12S rRNA, *coi* and *cytB* ML topologies, several *A*. *variegatum* samples from Zambia formed a subclade with moderate to high bootstrap support. Similarly to *A*. *hebraeum*, * A. variegatum* can be considered a generalist species in which high gene flow is expected. However, Matthee [63] noted that, unlike *A*. *hebraeum*, many generalist ectoparasite species show high levels of haplotype diversity. There are several potential reasons why species with similar ecology might exhibit different population genetics (especially with regard to mitochondrial markers), such as bottlenecks in evolutionary history, differential effects of migration or even the impact of vertically transmitted bacterial symbionts [64].

Phylogenetic analysis of the *Amblyomma* spp. indicated that all genes used in this study were adequate to differentiate between *A*. *eburneum*, *A*. *hebraeum* and the *A*. *variegatum*/*A*. *pomposum* complex. The different patterns that emerge between the phylogenetic trees could be a result of the differential mutation rates of each of the molecular markers [65]. Erster et al. [66] (and Koroiva and Santana [67]) evaluated the marker efficiency of 12S rRNA, 16S rRNA, *coi* and *cytB*, which were also used in the current study, and demonstrated that the mitochondrial markers *coi* and *cytB* were most suitable for intra- and inter-species analyses due to their high variability. Accordingly, Erster et al. [66] recorded barcode gaps of *12S* [0.00 ± 0.03], *coi* [0.03 ± 0.05] and *cytB* [0.04 ± 0.06], while the *16S* displayed a negative barcoding gap [−0.01 ± 0.06]. Furthermore, Vences et al. [68] reported that *cytB* was more variable than *coi*, allowing for clearer separation between closely related species of amphibians. Norris et al. [69] found that 16S rRNA was the least variable marker when comparing 12S rRNA and 16S rRNA markers for *Ixodes scapularis* population genetics. The 16S rRNA marker was also shown to be unable to differentiate between closely related species in the *Rhipicephalus sanguineus* group, including *Rhipicephalus turanicus* and *Rhipicephalus sanguineus*, and between *Rhipicephalus guilhoni*, *Rhipicephalus leporis* and *Rhipicephalus camicasi* [70]. The same limitation was observed for *Ixodes ricinus* and *Ixodes inopinatus*, which could not be differentiated between each other using 16S rRNA [71]. In the current study, *cytB* proved to be the most efficient single marker to differentiate between southern African *Amblyomma* spp. (Additional file 4: Dataset S3), while none of the markers in isolation allowed for clear discrimination between samples of the same species from different geographical regions.

In this context, a previous analysis of intraspecific variation of *A. variegatum* molecular markers concluded that genetic diversity was low in West Africa and in introduced Caribbean populations (i.e. nucleotide diversity of 0.02–0.25% for *12S*), with higher variation in East Africa (i.e. 0.65% for *12S*) [20]. Although the study did not apply the more variable markers used in the present work, the *12S* data alone suggest that southern populations of *A. variegatum* show greater genetic variation (nucleotide diversity of 1%) than elsewhere on the continent. Notably, a population genetic study of *A. variegatum* in Burkina Faso used microsatellites and concluded that effective population sizes were low at the village level when sampling domestic ruminants [72]. Similar approaches could also be applied to populations from southern Africa in future studies. Recently, mitochondrial markers were analysed to determine the genetic structure of another African *Amblyomma* sp., the elephant tick *A. tholloni*, on host populations in Kenya. The intra-specific variation at the *coi* locus was found to be low and of similar magnitude to that of *A. hebraeum* and *A. pomposum* in the current study (< 1%) [73].

Population genetic studies of *Amblyomma* spp. have been more extensive in the New World and offer important lessons for understanding intraspecific variation in the Afrotropical species. There are marked differences between the low intraspecific variation observed in some Neotropical species (e.g., *Amblyomma triste* [74] and *Amblyomma aureolatum* [75], where it was < 1% for mitochondrial markers) compared with others such as *Amblyomma ovale* [76, 77] and *Amblyomma mixtum* [33], where pairwise distances for concatenated mitochondrial markers can reach 3–5%. *Amblyomma cajennense*, which has an extensive distribution across subtropical and tropical regions of the Americas, constitutes a particularly interesting paradigm for the genus. Variation exceeding 8% at the whole mitogenome level between geographically and ecologically distinct populations of *A. cajennense* formed part of the evidence that was used to designate a species complex and formally describe its members as distinct species [78, 79]. The Afrotropical species await similar rigorous analyses using whole mitogenome data across the entirety of their range, as also highlighted in a recent study of *A. sparsum* in the *A. marmoreum* complex [61]. While the current study was not designed to address intraspecific population structure in depth, the concatenated phylogenetic analysis depicted clear differentiation between each of the analysed species. An important limitation was that the concatenated tree only had two representatives from *A*. *pomposum*. This was because not all loci had amplified successfully for each specimen.

Thus, on the basis of the phylogenetic analysis of the individual genes, the pairwise distance analyses and the ABGD analyses, we cannot conclude that *A*. *pomposum* and *A*. *variegatum* are distinct species. Literature on systematic work with *A*. *pomposum* is scarce, and currently there are no alternative methods to compare differences or similarities between these two species, except for morphological descriptions. No previous studies on the phylogenetic relationship of these two species have been conducted, and as discussed above, there is a scarcity of *A*. *pomposum* sequences available in GenBank. Kobayashi et al. [80] were the first authors who published the only sequences currently available for the *A*. *pomposum* on the GenBank database. They collected 15 *Amblyomma* spp. ticks and morphologically identified them as *A*. *variegatum* (*n* = 13) and *A*. *pomposum* (*n* = 2) with the use of Walker et al. [38]. They then conducted a phylogenetic analysis using the 16S rRNA gene. The *A*. *pomposum* that they identified clustered within the *A*. *variegatum* clade as in our study. Balinandi et al. [81] morphologically identified ticks they collected from Uganda as belonging to *A*. *pomposum* and *A*. *variegatum*; however, due to a lack of *A*. *pomposum* sequences at the time, phylogenetic analyses of the 16S rRNA gene clustered *A*. *pomposum* with *A*. *variegatum*. This discovery led the authors to believe that they had misidentified *A*. *variegatum* ticks as *A*. *pomposum*. Our 16S rRNA phylogenetic analysis also placed *A*. *pomposum* within the *A*. *variegatum* cluster. Moreover, based on our analysis of several genes, it is possible that Balinandi et al. [81] correctly identified their ticks as *A*. *pomposum*; however, since they did not upload any sequences nor depict the morphological discrepancy, no definite conclusion can be made on the basis of this information. Similarly, Barradas et al. [82] collected 116 ticks from the Huambo province in Angola. With the use of morphological identification using the key of Walker et al. [38], as well as molecular identification targeting the 12S rRNA and 16S rRNA genes, they identified 11 (10%) *Amblyomma* ticks as *A*. *variegatum*. As with the 16S rRNA analyses, the phylogenetics of 12S rRNA also indicated nesting of *A*. *pomposum* within *A*. *variegatum*. In our study, we collected from the same sites in Huambo and identified the *Amblyomma* spp. circulating in the area as *A*. *pomposum*. Our findings are also supported by Sili et al. [83], who identified all the *Amblyomma* spp. in this area as *A*. *pomposum*. On the basis of our morphological examinations at this geographic location, we believe that Barradas et al. [82] misidentified their *Amblyomma* spp. as *A*. *variegatum* instead of *A*. *pomposum*.

Although the phylogenetic analyses cannot differentiate between *A. variegatum* and *A. pomposum* currently, sufficient morphological features are documented to distinguish these species from each other (Table 1; Fig. 4). The other main factors defining a species are its ecological niche and reproductive behaviour [84]. Mayr [84] stated that a species can be described as “The segregation of the total genetic variability of nature into discrete packages, so called species, which are separated from each other by reproductive barriers, prevents the production of too great a number of disharmonious, incompatible gene combinations. This is the basic biological meaning of species and this is the reason why there are discontinuities between sympatric species”. A clear parapatric boundary was observed between *A*. *pomposum* and *A*. *variegatum*; however, a hypothesis of incipient speciation has yet to be tested. We suggest, alongside whole genome phylogenetic analysis, that mating and hybrid viability studies are performed between the two species to confirm their reproductive isolation.

## Conclusions

In this study,* cytB* was the most successful marker in differentiating between *A*. *eburneum*, *A*. *hebraeum*, *A*. *pomposum* and *A*. *variegatum*, closely followed by *coi*. The concatenated tree distinguished between *A*. *pomposum* and *A*. *variegatum*; however, the pairwise distance and ABGD analyses suggest there is insufficient evidence that *A*. *pomposum* and *A*. *variegatum* are distinct species. In our study, no *A*. *pomposum* ticks were collected in any country other than Angola, challenging the current distribution as proposed by Theiler and Salisbury [23], Walker et al. [38] and other current literature. On the basis of our findings, we suggest comparing whole mitochondrial genomes and several nuclear loci of *A*. *pomposum* with those of *A*. *variegatum* to determine whether these are distinct species, or whether *A*. *pomposum* is a subspecies of *A*. *variegatum*. Moreover, further investigations should also be conducted using mating and hybrid viability studies between the two species to determine whether they are reproductively isolated.

### Supplementary Information

Additional file 1. Text S1. Collection countries with the provinces, main locations, number of ticks collected, and the GPS co-ordinates for this stud.

Additional file 2: Table S1: Primer and PCR information. Table contains the primer target region, primer name, primer sequence, the expected product size, PCR master-mix volumes, annealing temperatures and reference article.

Additional file 3: GenBank accession numbers for the sequences used and obtained in this study.

Additional file 4: Dataset S3: Estimates of evolutionary divergence over sequence pairs between groups (interspecies p-distances). Values in red indicate the intraspecies p-distances.

Additional file 5: Estimates of Evolutionary Divergence between Sequences. The number of base differences per site from between sequences are shown. Standard error estimate(s) are shown above the diagonal. This analysis involved 143 nucleotide sequences. All ambiguous positions were removed for each sequence pair (pairwise deletion option). There were a total of 387 positions in the final dataset. Evolutionary analyses were conducted in MEGA11 [1].

Additional file 6: Estimates of Evolutionary Divergence between Sequences. The number of base differences per site from between sequences are shown. Standard error estimate(s) are shown above the diagonal. This analysis involved 111 nucleotide sequences. All ambiguous positions were removed for each sequence pair (pairwise deletion option). There were a total of 324 positions in the final dataset. Evolutionary analyses were conducted in MEGA11 [1].

Additional file 7: Estimates of Evolutionary Divergence between Sequences. The number of base differences per site from between sequences are shown. Standard error estimate(s) are shown above the diagonal. This analysis involved 141 nucleotide sequences. Codon positions included were 1st+2nd+3rd+Noncoding. All ambiguous positions were removed for each sequence pair (pairwise deletion option). There were a total of 651 positions in the final dataset. Evolutionary analyses were conducted in MEGA11 [1].

Additional file 8: Estimates of Evolutionary Divergence between Sequences. The number of base differences per site from between sequences are shown. Standard error estimate(s) are shown above the diagonal. This analysis involved 121 nucleotide sequences. Codon positions included were 1st+2nd+3rd+Noncoding. All ambiguous positions were removed for each sequence pair (pairwise deletion option). There were a total of 492 positions in the final dataset. Evolutionary analyses were conducted in MEGA11 [1].

Additional file 9. The number of base differences per site from between sequences are shown. Standard error estimate(s) are shown above the diagonal. This analysis involved 51 nucleotide sequences. All ambiguous positions were removed for each sequence pair (pairwise deletion option). There were a total of 1844 positions in the final dataset. Evolutionary analyses were conducted in MEGA11 [1].

Additional file 10: Fig. S1 Maximum likelihood (ML) analysis of the 12S rRNA gene. The ML analysis used the HKY + F + I model. Bootstrap values are indicated at each branch node; values under 70 were removed. Blue, pink, green and purple highlighted regions indicate *A*. *hebraeum*, *A*. *eburneum*, *A*. *variegatum* and *A*. *pomposum*, respectively. Species names are included with a three-letter country code and the sample name or GenBank accession number. Country codes: AGO, Angola; MOZ, Mozambique; NGA, Nigeria; STP, São Tomé and Príncipe; SWZ, Eswatini; ZAF, South Africa; ZM, Zambia; and ZWE, Zimbabwe. GenBank reference strains are marked with ✦

Additional file 11: Fig. S2 Maximum likelihood (ML) analysis of the 16S rRNA gene. The ML analysis used the HKY + F + R2 model. Bootstrap values are indicated at each branch node; values under 70 were removed. Blue, pink, green and purple highlighted regions indicate *A. hebraeum*, *A. eburneum*, *A. variegatum*, and *A. pomposum*, respectively. Species names are included with a three-letter country code and the sample name or GenBank accession number. Country codes: AGO, Angola; KEN, Kenya; MOZ, Mozambique; UGA, Uganda; ZAF, South Africa; ZM, Zambia; and ZWE, Zimbabwe. GenBank reference strains are marked with ✦

Additional file 12: Fig. S3 Maximum likelihood (ML) analysis of the *coi* gene. The ML analysis used the TPM2 + F + G4 model. Bootstrap values are indicated at each branch node; values under 70 were removed. Blue, pink, green and purple highlighted regions indicate *A. hebraeum *, *A. eburneum*, *A. variegatum* and *A. pomposum*, respectively. Species names are included with a three-letter country code and the sample name or GenBank accession number. Country codes: AGO, Angola; CMR, Cameroon; GNB. Guinea-Bissau; KEN, Kenya; MOZ, Mozambique; SEN, Senegal; SWE, Eswatini; ZAF, South Africa; ZMB, Zambi; and ZWE, Zimbabwe. GenBank reference strains are marked with **

Additional file 13: Fig. S4 Maximum likelihood (ML) analysis of the *cytB* gene. The ML analysis used the HKY + F + G4 model. Bootstrap values are indicated at each branch node; values under 70 were removed. Blue, pink, green and purple highlighted regions indicate *A. hebraeum *, *A. eburneum*, *A. variegatum* and *A. pomposum*, respectively. GenBank reference strains are marked with **

## Data Availability

The data supporting the main findings of this study are included in the manuscript and its supplementary files. Sequences generated in this study were deposited in GenBank and the accession numbers are available in Additional file 3: Dataset S2.

## Abbreviations

ABGD: Automatic Barcode Gap Discovery
BI: Bayesian inference
CAF: Central Analytical Facility
ESS: Estimates sample size
ML: Maximum likelihood
OTUs: Operational taxonomic units
SE: Standard error
SNP: Single nucleotide polymorphisms
