# Supplementary material for: Intra- and interspecific variation of Amblyomma ticks from southern Africa
Source: Parasit Vectors. 2024 Aug 28;17:364. doi: 10.1186/s13071-024-06394-3 (PMC11351087; doi:10.1186/s13071-024-06394-3)
Supplement: Supplementary file 1 — Additional file 1. Text S1. Collection countries with the provinces, main locations, number of ticks collected, and the GPS co-ordinates for this stud. [file 13071_2024_6394_MOESM1_ESM.docx]

**Additional File 1: Text S1**. Collection countries with the provinces, main locations, number of ticks collected, and the GPS co-ordinates for this study

| Sample Country | Sampling Provinces | Main Locations | Number of collected ticks | GPS locations | |
| --- | --- | --- | --- | --- | --- |
|  |  |  |  | Latitude (S) | Longitude (E) |
| Angola | Cunene | Chiulo | 1 | 16.51886 | 14.84050 |
|  |  | Xangongo | 1 | 16.77291 | 14.98890 |
|  | Benguela | Benguela | 32 | 15.875966 | 14.079374 |
|  | Huíla | Chibemba | 7 | 15.748619 | 14.059498 |
|  | Huambo | Caala | 132 | 13.14762 | 15.67408 |
|  |  | Tchicala | 18 | 12.72992 | 16.08131 |
|  | Moxico | Lucusse | 259 | 12.50352 | 20.89781 |
| Mozambique | Maputo | Magude | 60 | 25.00420 | 32.73285 |
|  |  | Gala | 102 | 26.62304 | 32.84057 |
|  |  | Moamba | 182 | 24.97277 | 32.21438 |
|  |  | Manisha | 293 | 25.45906 | 32.60032 |
|  | Gaza | Bilene | 455 | 24.84542 | 33.08407 |
|  |  | Mabalane | 285 | 23.84720 | 32.62291 |
|  |  | Chongoene | 391 | 24.88339 | 32.81821 |
|  |  | Guija | 294 | 24.39799 | 32.14991 |
|  | Inhambane | Inhassoro | 85 | 21.59656 | 35.13190 |
|  |  | Inharrime | 148 | 24.24463 | 35.03220 |
|  |  | Hermoine | 465 | 23.94058 | 35.09099 |
|  |  | Massinga | 307 | 23.42132 | 35.39648 |
|  |  | Mambone | 113 | 21.08917 | 34.94773 |
|  |  |  | 53 | 21.12336 | 34.79602 |
|  |  |  | 176 | 21.11465 | 34.91785 |
|  | Manica | Cupenha | 40 | 19.44223 | 33.32338 |
|  |  | Penhalonga | 180 | 18.87350 | 32.77740 |
|  |  | Macate | 80 | 19.19042 | 33.43880 |
|  | Sofala | Chaideia | 361 | 19.56059 | 34.26601 |
|  |  |  | 106 | 19.55215 | 34.36478 |
|  |  |  | 4 | 19.48321 | 34.58872 |
|  |  |  | 16 | 19.69299 | 34.79889 |
|  |  | Mungari | 1040 | 18.39371 | 35.59856 |
| South Africa | Gauteng | Rust de Winter | 100 | 25.1136 | 28.4603 |
|  | Kwazulu-Natal | KwaDukuza | 84 | 29.1769 | 31.2822 |
|  |  | Ulundi | 120 | Not recorded | |
|  |  | Escourt Inkosi Langalibalele | 140 | 28.5424 | 30.1019 |
|  | Limpopo | Lepelle Nkumpi | 102 | 24.2342 | 29.8276 |
|  |  | Thabazimbi | 136 | 24.1183 | 30.1110 |
|  | Mpumalanga | Albert Luthuli | 59 | 26.1089 | 30.9441 |
|  |  | Thembisile Hani | 88 | 25.3072 | 29.0750 |
|  |  | Bushbuckridge | 350 | Not recorded | |
| Zambia | Western | Mongu | 32 | 15.38914 | 23.20239 |
|  |  |  | 197 | 15.45754 | 23.39825 |
|  |  |  | 78 | 15.18766 | 22.83576 |
|  | Lusaka | Lusaka | 42 | 15.46060 | 28.77422 |
|  |  |  | 54 | 15.451087 | 27.83406 |
|  | Eastern | Petauke | 48 | 14.39572 | 31.38466 |
|  |  |  | 110 | 14.44079 | 31.38581 |
|  |  |  | 6 | 14.35060 | 31.37484 |
| Zimbabwe | Midlands | Gokwe south | 23 | 18.316667 | 28.782333 |
|  |  | Kwekwe | 14 | 18.427710 | 29.387663 |
|  |  | Shurugwi | 10 | 19.825537 | 30.424108 |
|  | Masvingo | Chiredzi | 53 | 22.27023 | 31.22882 |
|  |  |  | 39 | 22.29934 | 31.1418 |
|  |  | Mkienezi | 74 | 21.21667 | 30.3951 |
|  | Mashonaland West | Mazokie | 9 | 17.083 | 30.696 |
|  | Matabeleland South | Zvishavane | 40 | 20.053 | 29.5997 |
|  |  |  | 51 | 20.11234 | 29.9144 |
|  |  |  | 32 | 20.0076 | 29.5629 |
