# Supplementary figures and images for: Intra- and interspecific variation of Amblyomma ticks from southern Africa
Source: Parasit Vectors. 2024 Aug 28;17:364. doi: 10.1186/s13071-024-06394-3 (PMC11351087; doi:10.1186/s13071-024-06394-3)

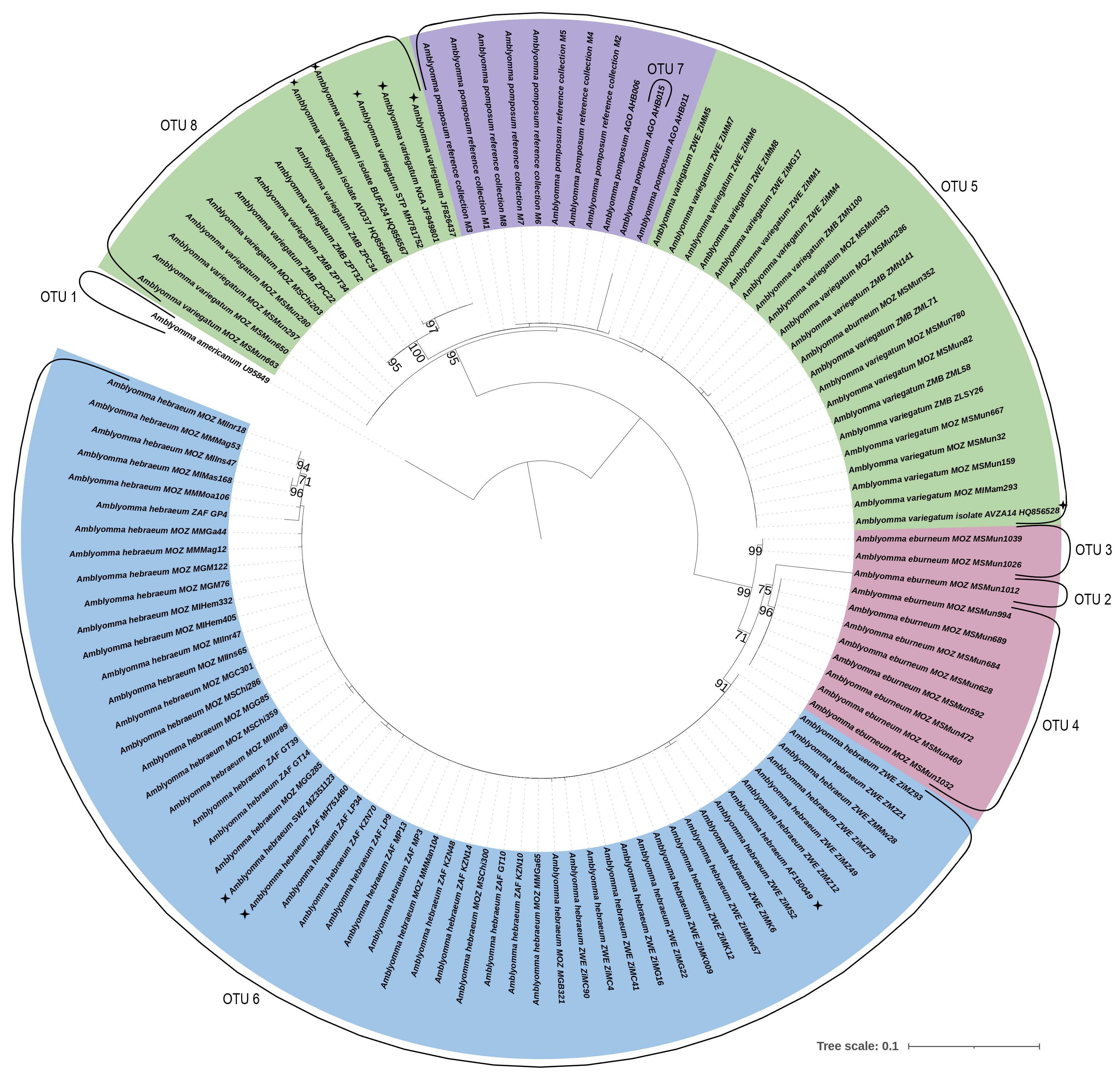

Supplement: Supplementary file 10 — Additional file 10: Fig. S1 Maximum likelihood (ML) analysis of the 12S rRNA gene. The ML analysis used the HKY + F + I model. Bootstrap values are indicated at each branch node; values under 70 were removed. Blue, pink, green and purple highlighted regions indicate A. hebraeum, A. eburneum, A. variegatum and A. pomposum, respectively. Species names are included with a three-letter country code and the sample name or GenBank accession number. Country codes: AGO, Angola; MOZ, Mozambique; NGA, Nigeria; STP, São Tomé and Príncipe; SWZ, Eswatini; ZAF, South Africa; ZM, Zambia; and ZWE, Zimbabwe. GenBank reference strains are marked with ✦ [file 13071_2024_6394_MOESM10_ESM.jpg]

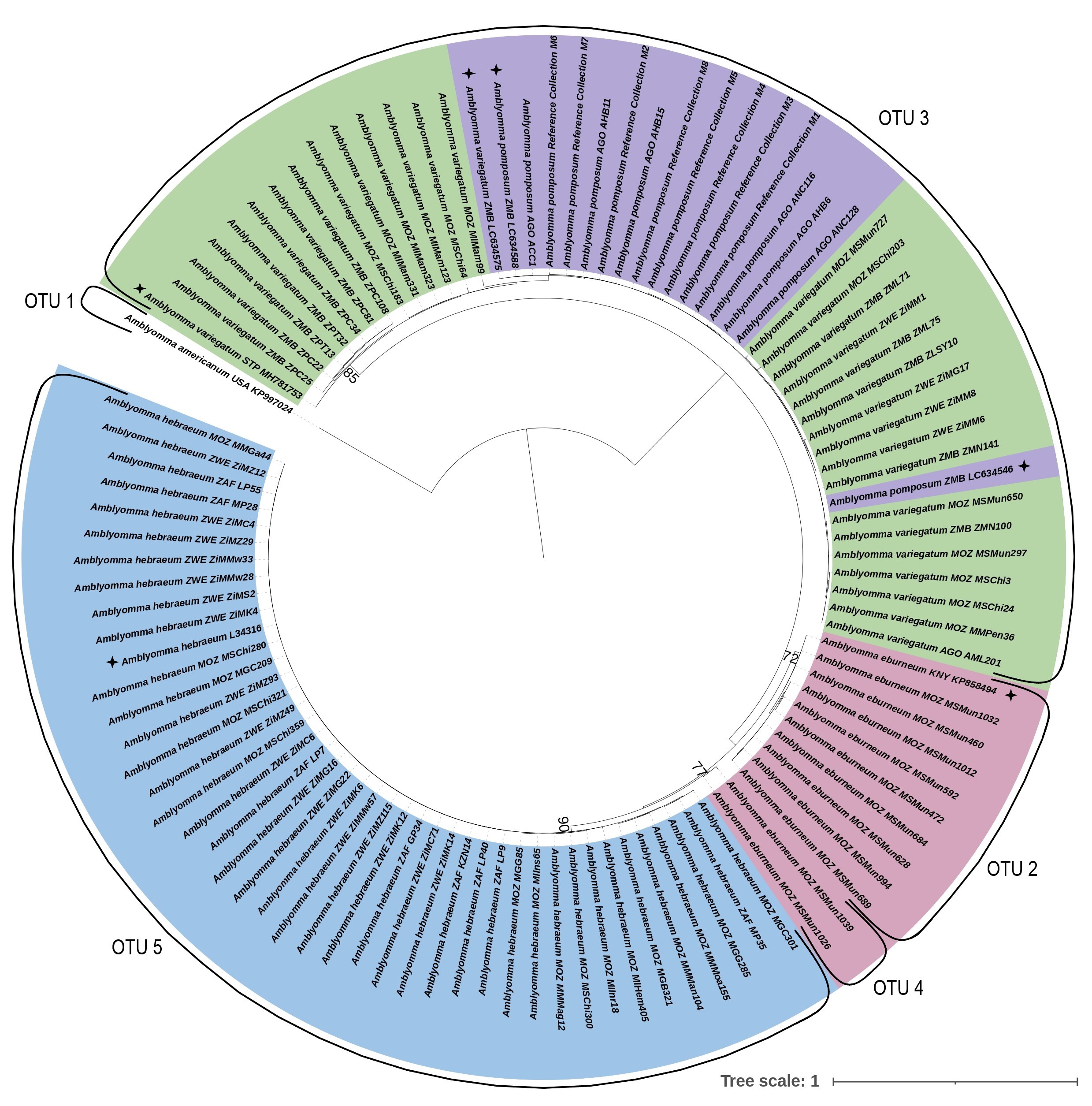

Supplement: Supplementary file 11 — Additional file 11: Fig. S2 Maximum likelihood (ML) analysis of the 16S rRNA gene. The ML analysis used the HKY + F + R2 model. Bootstrap values are indicated at each branch node; values under 70 were removed. Blue, pink, green and purple highlighted regions indicate A. hebraeum, A. eburneum, A. variegatum, and A. pomposum, respectively. Species names are included with a three-letter country code and the sample name or GenBank accession number. Country codes: AGO, Angola; KEN, Kenya; MOZ, Mozambique; UGA, Uganda; ZAF, South Africa; ZM, Zambia; and ZWE, Zimbabwe. GenBank reference strains are marked with ✦ [file 13071_2024_6394_MOESM11_ESM.jpg]

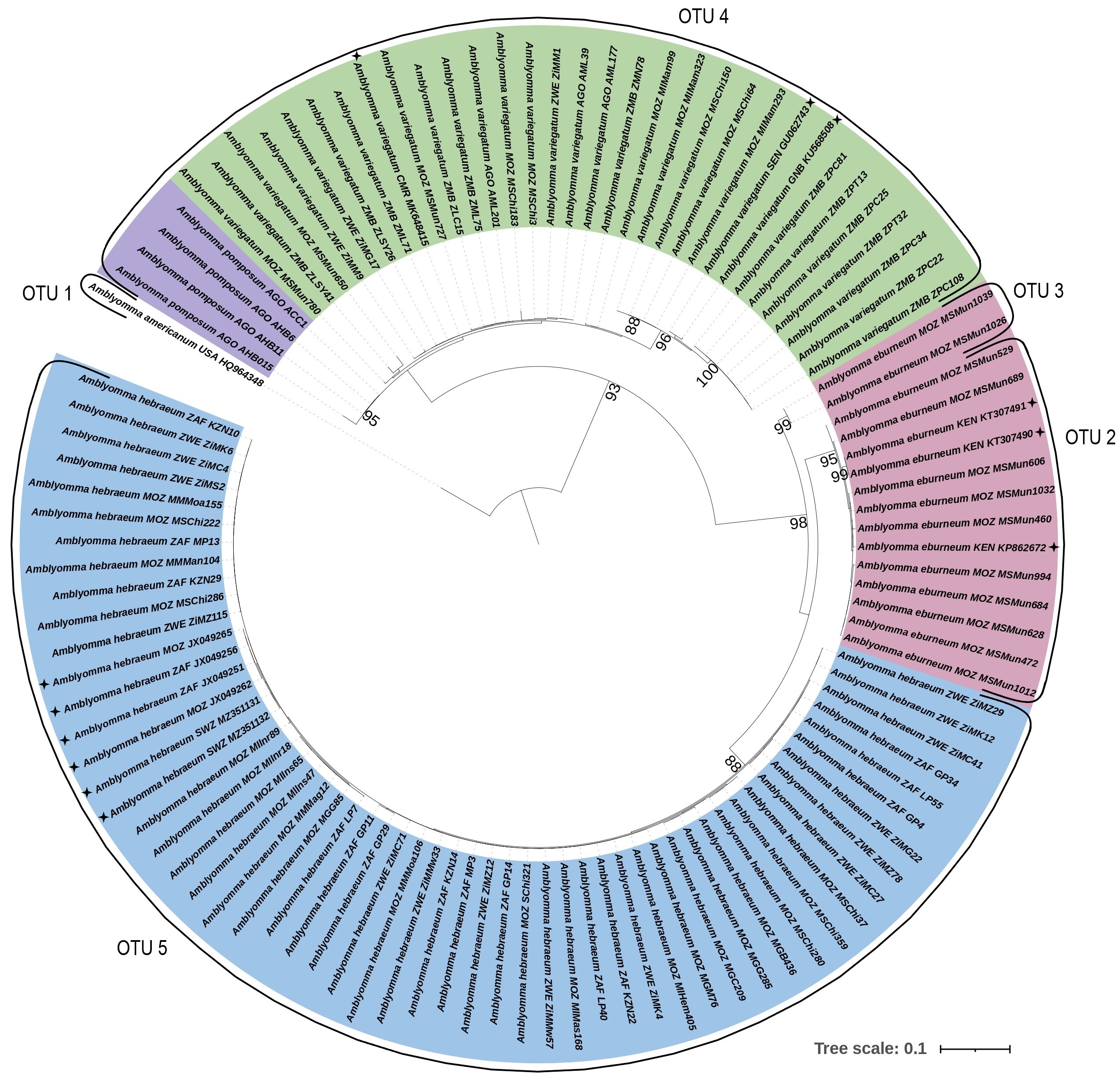

Supplement: Supplementary file 12 — Additional file 12: Fig. S3 Maximum likelihood (ML) analysis of the coi gene. The ML analysis used the TPM2 + F + G4 model. Bootstrap values are indicated at each branch node; values under 70 were removed. Blue, pink, green and purple highlighted regions indicate A. hebraeum , A. eburneum, A. variegatum and A. pomposum, respectively. Species names are included with a three-letter country code and the sample name or GenBank accession number. Country codes: AGO, Angola; CMR, Cameroon; GNB. Guinea-Bissau; KEN, Kenya; MOZ, Mozambique; SEN, Senegal; SWE, Eswatini; ZAF, South Africa; ZMB, Zambi; and ZWE, Zimbabwe. GenBank reference strains are marked with ** [file 13071_2024_6394_MOESM12_ESM.jpg]

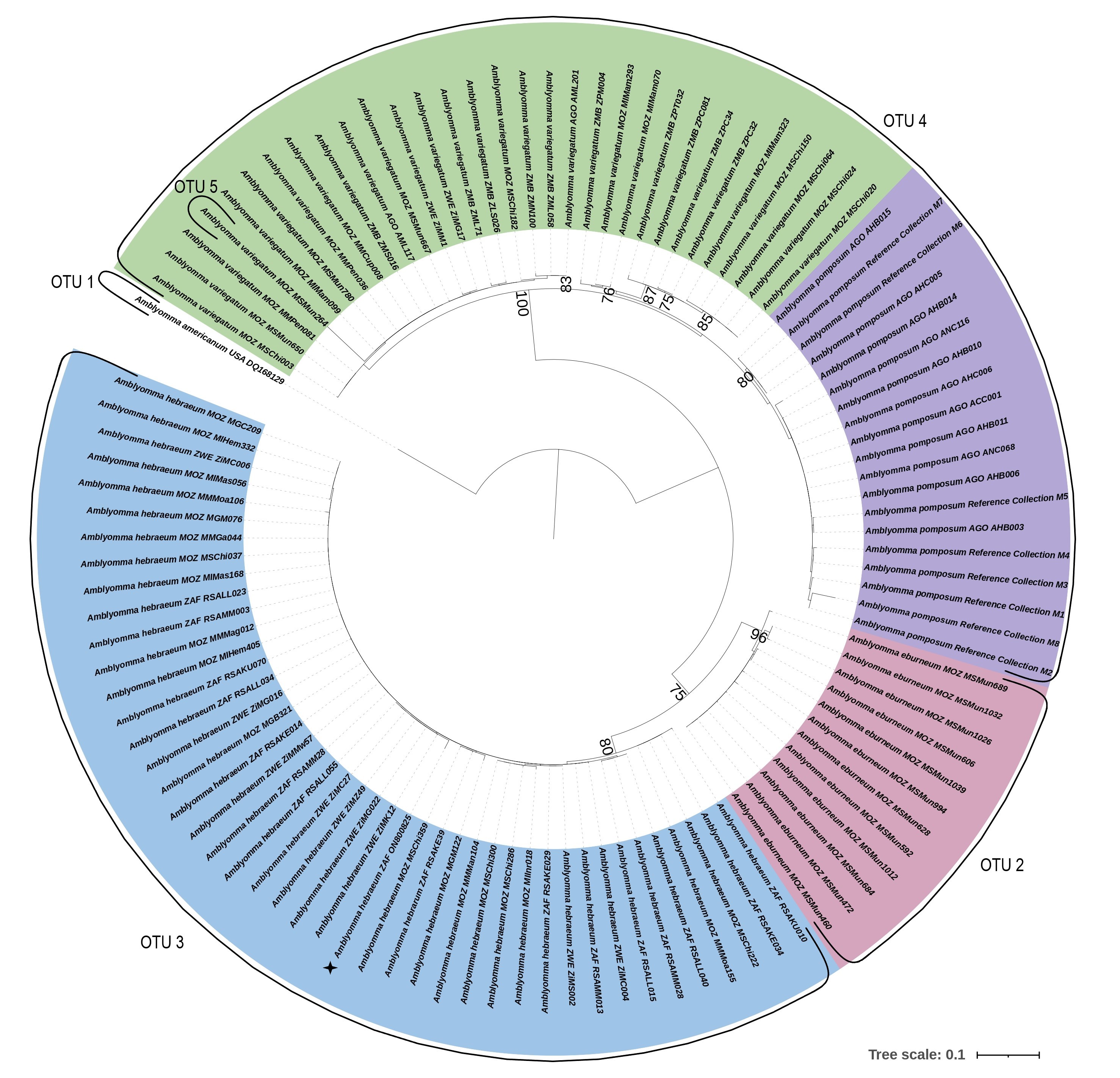

Supplement: Supplementary file 13 — Additional file 13: Fig. S4 Maximum likelihood (ML) analysis of the cytB gene. The ML analysis used the HKY + F + G4 model. Bootstrap values are indicated at each branch node; values under 70 were removed. Blue, pink, green and purple highlighted regions indicate A. hebraeum , A. eburneum, A. variegatum and A. pomposum, respectively. GenBank reference strains are marked with ** [file 13071_2024_6394_MOESM13_ESM.jpg]
